# Preventing the Reintroduction of Malaria in Mauritius: A Programmatic and Financial Assessment

**DOI:** 10.1371/journal.pone.0023832

**Published:** 2011-09-02

**Authors:** Allison Tatarsky, Shahina Aboobakar, Justin M. Cohen, Neerunjun Gopee, Ambicadutt Bheecarry, Devanand Moonasar, Allison A. Phillips, James G. Kahn, Bruno Moonen, David L. Smith, Oliver Sabot

**Affiliations:** 1 Clinton Health Access Initiative, Boston, Massachusetts, United States of America; 2 The Global Health Group, University of California San Francisco, San Francisco, California, United States of America; 3 Ministry of Health and Quality of Life, Port Louis, Mauritius; 4 National Department of Health, Pretoria, South Africa; 5 Department of Epidemiology and Biostatistics, University of California San Francisco, San Francisco, California, United States of America; 6 Emerging Pathogens Institute and Department of Biology, University of Florida, Gainesville, Florida, United States of America; Kenya Medical Research Institute - Wellcome Trust Research Programme, Kenya

## Abstract

Sustaining elimination of malaria in areas with high receptivity and vulnerability will require effective strategies to prevent reestablishment of local transmission, yet there is a dearth of evidence about this phase. Mauritius offers a uniquely informative history, with elimination of local transmission in 1969, re-emergence in 1975, and second elimination in 1998. Towards this end, Mauritius's elimination and prevention of reintroduction (POR) programs were analyzed via a comprehensive review of literature and government documents, supplemented by program observation and interviews with policy makers and program personnel. The impact of the country's most costly intervention, a passenger screening program, was assessed quantitatively using simulation modeling.

On average, Mauritius spent $4.43 per capita per year (pcpy) during its second elimination campaign from 1982 to 1988. The country currently spends $2.06 pcpy on its POR program that includes robust surveillance, routine vector control, and prompt and effective treatment and response. Thirty-five percent of POR costs are for a passenger screening program. Modeling suggests that the estimated 14% of imported malaria infections identified by this program reduces the annual risk of indigenous transmission by approximately 2%. Of cases missed by the initial passenger screening program, 49% were estimated to be identified by passive or reactive case detection, leaving an estimated 3.1 unidentified imported infections per 100,000 inhabitants per year.

The Mauritius experience indicates that ongoing intervention, strong leadership, and substantial predictable funding are critical to consistently prevent the reestablishment of malaria. Sustained vigilance is critical considering Mauritius's enabling conditions. Although the cost of POR is below that of elimination, annual per capita spending remains at levels that are likely infeasible for countries with lower overall health spending. Countries currently embarking on elimination should quantify and plan for potentially similar POR operations and costs.

## Introduction

Recently, a growing number of countries have experienced dramatic reductions in malaria transmission and have set short-term goals for elimination [1]. Among this group are a number of countries in sub-Saharan Africa and other regions where baseline malaria transmission is high [1]. Several countries, including Morocco, Oman, and the United Arab Emirates, have recently achieved elimination and others are on the verge of doing so [2]. The recent surge of interest in and pursuit of elimination requires a close examination of post-elimination, or prevention of reintroduction (POR), activities. While recent recommendations suggest that countries should thoroughly assess the feasibility of preventing reintroduction prior to embarking on a serious elimination effort [3], many outstanding questions surrounding malaria elimination and POR remain. What is the cost structure of successful elimination and POR programs? Can malaria-free status be maintained in areas with an efficient vector and frequent importation of new cases? What is an effective combination of interventions to sustain elimination?

Despite the fact that several countries have been actively preventing the reintroduction of malaria over the past several decades [2], there continues to be a dearth of evidence about this phase. POR was considered only superficially during the Global Malaria Eradication Program (GMEP) since a global campaign by definition implied that importation and resurgence were not of significant concern. Since then, most evidence generated has focused on control in high endemic areas or the process of interrupting transmission [4], [5]. As a result, only a limited empirical foundation is available today to guide strategic decision-making in countries that may successfully achieve elimination without the benefit of their neighbors and the wider malaria endemic world doing the same.

To help close this evidence gap, the elimination and prevention of reintroduction experience on the island nation of Mauritius was closely analyzed. The Republic of Mauritius consists of several reefed islands in the Indian Ocean, including the larger populated islands of Mauritius and Rodrigues with a total population of 1,288,000 in 2009 [6]. The islands experience subtropical climate year round and heavy rainfall from December to May during the hot, wet summer with frequent and often destructive cyclones [7]. Total expenditure on health as a percentage of GDP in 2009 was 5.7% [6].

*Plasmodium vivax* and *Plasmodium falciparum* and their vectors, *Anopheles funestus* and *Anopheles arabiensis*, were most likely imported into Mauritius by ships with slaves and indentured laborers arriving from malaria-endemic East Africa and South Asia during colonization from early 1800 to 1860 [8]. In 1867, a violent malaria epidemic erupted in Mauritius that resulted in 40,000 deaths of a population 330,000, with 6,000 deaths occurring in just one month in urban Port Louis [9]. Mauritius was globally notorious for its malariousness after the epidemic, making the achievement of elimination that much more remarkable more than 100 years later [8].

Mauritius's experience is well suited to generate lessons for the current wave of countries today that are pursuing or considering elimination. It is one of only two sub-Saharan African countries to have fully eliminated malaria and has historically faced malaria transmission equivalent to many mainland countries [10]. Mauritius, a country that interrupted local transmission in 1969, saw it reemerge in 1975, and once again ended transmission in 1998, continues to receive high volumes of travelers from malaria endemic countries despite its relative isolation.

This paper examines the history of malaria elimination in Mauritius, with a particular focus on the country's POR programs. The composition and costs of both elimination and POR programs are analyzed and the impact of its single most costly component, a passenger screening program, is examined quantitatively to provide evidence on its effectiveness and cost-efficiency. Finally, recommendations are presented based on the Mauritius experience to inform decision-making in other countries embarking on malaria elimination.

## Methods

### Literature review and interviews

To identify all available information on the history of malaria epidemiology, control, and elimination in Mauritius, a systematic literature review was conducted. PubMed (United States National Library of Medicine), OVID (Ovid Technologies, Inc.), and Google Scholar databases were searched using the keywords “malaria”, “Mauritius”, and “eradication” or “elimination.” Relevant citations contained in resulting publications were also included, as well as published government and WHO reports and digitized books. In addition, all gray literature available at the National Archives, Health Statistics Unit, Mauritius Institute of Health, and Communicable Disease Control Unit (CDCU) of the Ministry of Health and Quality of Life was searched for reference to malaria, malaria control, or elimination. Only literature dated from 1860, the time of emergence of malaria in Mauritius, was included in the review. All narratives, health statistics, and financial budgets related to malaria in Mauritius were extracted from this subset of reports and publications and compiled for analysis by AT.

Direct observation of ongoing surveillance and vector control activities furnished additional insights, and visits to major implementing institutions in Mauritius and the ports of entry allowed closer examination of the passenger screening program. Further information was collected through approximately 50 interviews using semi-structured questionnaires with key technical experts, policy makers, and operational personnel from past and present malaria programs. All individuals were purposively selected based on their professional affiliation in public health, most of whom had current or past involvement in malaria financing, program management, or implementation. Information was verified through document review, and, when possible, from additional individuals with identical rank and responsibility.

### Program costing

All identified costs from budgets, technical reports, and program reviews were allocated to specific activities within four main intervention categories – surveillance and diagnosis; treatment; prevention; and management. Within each activity, costs were classified as personnel, consumables, capital equipment, training, or services.

Comprehensive costing data were available for both elimination campaigns, 1948–1951 and 1982–1988. Costs were also available for 1960–1961, 1990–1991, and 2008. Although local transmission was not interrupted until 1968 and re-interrupted until 1998, interventions and strategies in 1960 and 1990 were very similar to those during POR. Malaria incidence had virtually reached zero during these years [11], [12] and strategies were in place that continued until reemergence (1975) [13] and through the early 1990s [14]. Therefore, costs for these two years and for 2008 are considered representative of POR and are analyzed as such in this paper.

Personnel costs for 1949–1961 were collected from the National Accounts and the Mauritius Blue Book of budget salary estimates [15], [16] and supplemented by technical reports [7], [17]. These same sources for later years omitted substantial expenditures, i.e., travel and overtime that contributed between 20% and 50% to personnel costs beyond basic salary [18], [19]. Thus, complete personnel costs for the 1980s were extrapolated based on fiscal year 1990/1991 when more comprehensive data were available [18], [20] verified by program staff employed at the time using an average annual inflation rate between 1982/1983 and 1987/1988 of 4.7% [21] and assuming a constant annual change in salaries. Costs for 2008 were collected from a number of finance and implementing institutions within the Ministry of Health and Quality of Life, including the Finance Section, Communicable Disease Control Unit, Central Health Laboratory Malaria Section, Vector Biology and Control Division, and the Procurement Section.

This analysis included only malaria-specific costs (i.e. excluding general health system resources). Thus time spent on malaria-related activities per person per grade was estimated since the malaria program was integrated into the health system at various points throughout elimination and POR. Two methods were used to identify all personnel costs: interviews with current and former staff on the average number of hours or days spent on malaria each week, and review of narratives in technical reports from the recent elimination campaign.

Costs beyond personnel were derived from reports of actual expenditures and prospective budgets. Approximately 40% of these costs for elimination was actual expenditure reported subsequent to implementation, while remaining costs were prospective estimates found in program budgets. All costing data for the current POR program includes actual expenditure.

Straight-line amortization was used for capital equipment and all costs were apportioned among activities based on the judgment of local staff for recent costs or reports from past programs. All costs were indexed to the year 2008 using local GDP deflators for Mauritius [22] and then converted to USD [23], [24].

### Assessing the impact of surveillance measures

A quantitative analysis was conducted to understand the impact of the interventions implemented and estimate the risk averted by the current POR program in Mauritius. The risk of renewed local transmission following elimination is dependent upon two principal factors: the rate at which new infections are imported, and the probability of those infections leading to onwards transmission [25]. Estimating the probability of onward transmission – best expressed in terms of the basic reproductive number, R_0_ [26]–is made extremely challenging in Mauritius by the long absence of malaria transmission from the islands; relying on estimates from many decades in the past is problematic due to changes in socioeconomic status, environment, and vectors [27]. As such, a range of values for this parameter was considered for this analysis.

Existing comprehensive surveillance data enabled detailed consideration of the rate at which infections are imported. The number of infections detected by current measures was summed from proactive case detection (passenger screening), passive case detection, and reactive case detection records [28] [see Table 1 for definitions]. Some infections may have been missed by all of these case detection methods so the number of unidentified cases was estimated. Passengers arriving in Mauritius are screened proactively if they are febrile and/or have a recent travel history of being in a malaria endemic country [31]. Malaria cases thus may be missed at the ports of entry if they do not display fever, if their travel history is incomplete or inaccurate, or if the sensitivity of microscopy is imperfect. Cases may then be identified through passive or reactive case detection. Combining assumptions about each of these three variables – the fraction of cases that are asymptomatic, the fraction that have incomplete travel histories, and the sensitivity of microscopy – with records of the fraction of incoming passengers who meet the criteria for screening but who are either untraceable or do not require testing (only passengers who are symptomatic at some point during the 42-day surveillance period are tested), permitted calculation of the number of cases missed by this screening approach according to the following equation, derived from a simple decision-tree model:
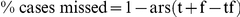
Where

a = the fraction of those who meet the passenger screening criteria who cannot be located for follow up (5.5%, per 2008 data [32])

r = the fraction of those who meet the passenger screening criteria who were located and followed up but not tested (75.4%, per 2008 data [32])

s = the percent sensitivity of microscopy (a range of values estimated from literature [33])

t = the unknown fraction of incoming passengers with malaria for whom an accurate travel history is recorded

f = the unknown fraction of incoming passengers with malaria who are febrile

Because values s, t and f are unknown, distributions for each were assumed. The fraction of cases with fever was allowed to vary across a wide uniform distribution of 10–80% to reflect the great uncertainty in this estimate, while the fraction of infected individuals with complete travel histories was assumed to follow a narrower uniform distribution ranging from 80–100%; sensitivity analyses demonstrated that the model was largely insensitive to both of these values. Finally, the sensitivity of microscopy was estimated to follow a normal distribution with mean 70% and SD = 10%. The mean value was calculated by combining estimates of microscopy sensitivity from a recent review [33] with a weighted average of the prevalence of malaria at the origin of recorded imported cases from 2005–2008. Values were picked at random for each variable from these three distributions in 99,999 Monte Carlo simulations, and the average fraction of cases missed and its 95% confidence interval were calculated from the resulting distribution of outcomes.

**Table 1:** Surveillance definitions.

**Passive case detection**	Involves a system in which data are routinely received by a central health authority based on a set of rules and laws that need a health-care provider or health facility to report some diseases or disorders on an ongoing basis and at specific intervals [29]
**Reactive case detection**	Is triggered whenever a case is identified by passive case detection and involves visiting the household of the locally acquired case, screening family members, and screening neighbors within a defined radius [30]
**Proactive case detection (e.g. passenger screening)**	Involves the screening of focal populations without the trigger of a passively identified case based on the knowledge that transmission is more likely during some periods of the year, in specific high-risk groups, or in target geographical areas [30]

### Simulating the importance of missed cases for prevention of reintroduction

The impact of current passenger screening on preventing reemergence of malaria in Mauritius was examined through application of an individual-based, spatially-explicit, stochastic simulation model that has been described elsewhere [34]. This model requires specification of the importation rate per 1,000 inhabitants per year; R_C_, the number of malaria cases resulting from each case given ongoing control [35]; and the fraction of cases rapidly identified and treated by the health system. First, transmission was simulated using an importation rate derived from the number of infections estimated to be missed by the current screening program, and second, with a higher importation rate corresponding to the expected rate if no passenger screening was conducted. Because R_C_ is unknown for Mauritius, this parameter was varied in each case from 0–0.5, a range of values consistent with the island's demonstrated lack of local transmission over the past decade. The number of infections estimated to be missed by both the passenger screening program and subsequent passive and reactive case detection was used to derive the case detection rate. For each scenario, 1,000 1-year simulations were run, and the fraction of those iterations in which indigenous (that is, not imported nor introduced) malaria occurred was tallied to produce an annual risk.

## Results

The review identified approximately 543 publications on malaria in Mauritius in the peer-reviewed literature as well as Government of Mauritius, Ministry of Health and Quality of Life, and World Health Organization reports on the incidence of malaria, financing of the control program, and coverage with interventions for the years 1855 to 2008. From these, a subset of 61 particularly comprehensive accounts of malaria incidence and control were selected for summary here. This literature includes 12 publications and/or reports providing comprehensive data on reported malaria incidence over time; six which provide a complete accounting of costs for the years 1948–1951, 1962, 1981–1988, 1992, and 2008; 19 Ministry of Health reports detailing the interventions implemented from 1945 to 2008; five WHO reports on the most recent elimination campaign; and 19 publications on the global malaria elimination dialogue and costing methods.

Thirty percent of the semi-structured in-depth interviews took place with high level administrative and technical personnel in the Ministry of Health and Quality of Life; 50% with implementing officers at both the national and district levels; 15% with retired experts formerly involved in communicable disease control; and the remaining with officers from external agencies. Respondents provided information on roles and responsibilities of the human resource infrastructure for current and past malaria programs and on time-spent on malaria-related activities that supported the costing analysis conducted in this paper. Respondents further provided narratives on the challenges and successes of past and current malaria programs.

### Elimination, 1948–1968

Following the introduction of malaria to Mauritius in the 1860s [36], the disease became highly endemic in the island, with parasite rates (65% in school children and 38% in the general population in 1946 [10]) similar to those in many high-burden mainland countries [2]. *Anopheles funestus* was a highly efficient vector during this period but has not been detected since cessation of the first elimination campaign from 1948–1952 with wide scale use of DDT (dichlorodiphenyltrichloroethane) [37]. Since then, *An. arabiensis* remains the only malaria vector in Mauritius.

**Figure 1:**
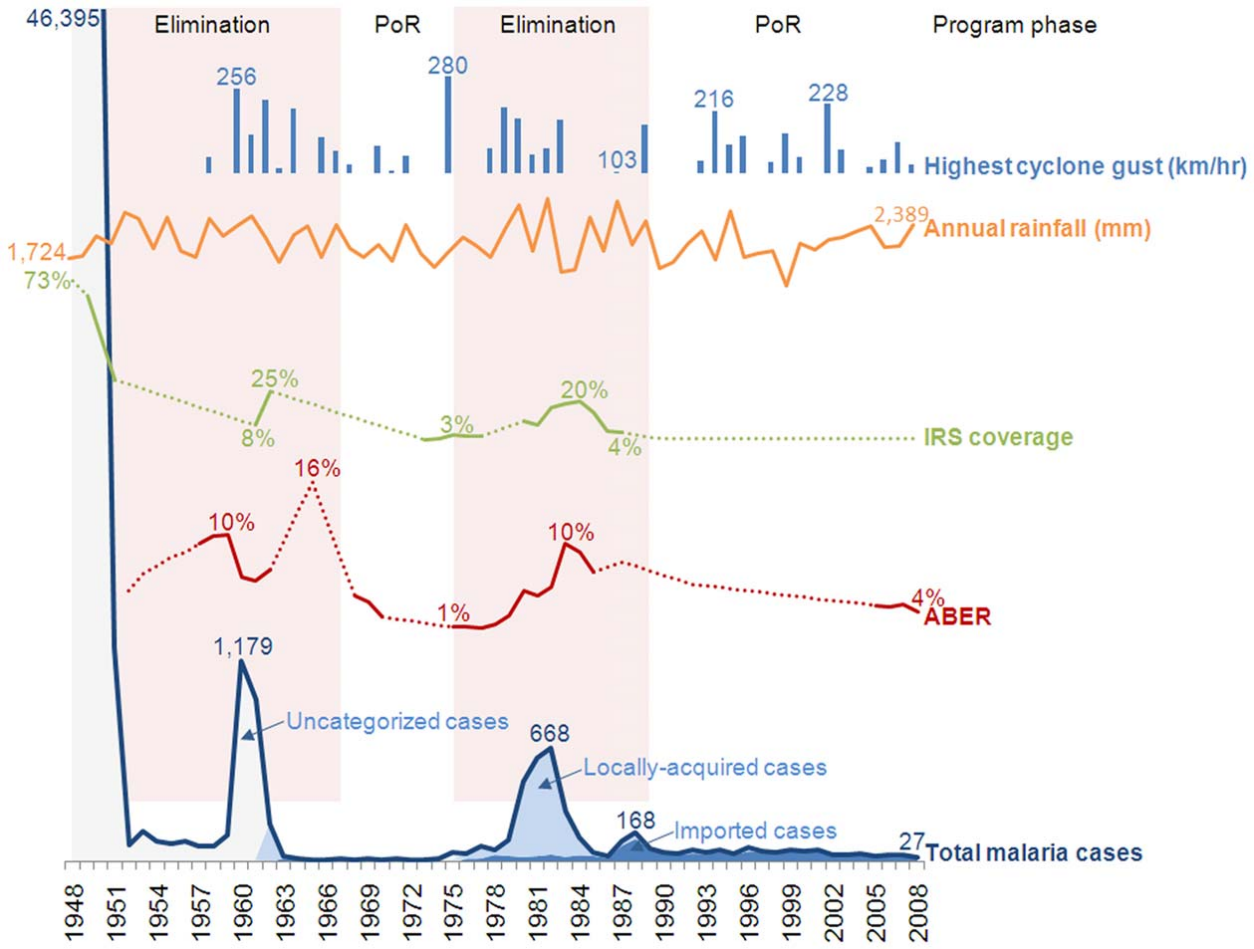
Key indicators throughout elimination and prevention of reintroduction in Mauritius, 1948–2008. Abbreviations: ABER - Annual Blood Examination Rate, IRS - Indoor Residual Spraying, POR - Prevention of Reintroduction.

Figure 1 describes epidemiological, meteorological and programmatic trends between 1948 and 2008. From 1948 to 1952, Mauritius conducted an aggressive campaign to eliminate malaria through indoor residual spraying (IRS) with DDT – the strategy deployed by the GMEP [8] – protecting more than 70% of the island. In just four years, malaria incidence dropped from 105 cases per 1,000 population to 2.6 cases per 1,000 population. The program subsequently shifted from blanket spraying to targeted spraying of active and residual foci. Surveillance was strengthened by establishing a Mobile Malaria Squad, the country's first active case detection system that reactively screened contacts of malaria cases and proactively conducted fever surveys [17]. A spike in cases in 1960 was likely the result of the introduction of this mobile squad and a change in surveillance strategy when screening shifted from mass blood to fever surveys, a more sensitive system for case detection. Local malaria transmission was interrupted in 1969, and Mauritius received malaria-free certification by the WHO in 1973 [38].

### Prevention of reintroduction, 1969–1974

In 1968, the malaria unit was integrated into the preventive division of the health system [39], as was the malaria laboratory in 1969 [40], which resulted in reduction in full-time equivalents (FTEs) dedicated to malaria-related activities [13]. To reduce the risk of resurgence, the program continued to conduct vector control activities between 1969 and 1974, including routine island-wide larviciding and DDT spraying at the ports of entry initially every three (1968–1970) or six (1971–1974) months [40]–[44].

The proactive case detection program that was initiated in 1960 continued during POR and included two key interventions: 1) fever surveys in residual transmission foci, and 2) a passenger screening program to manage the continued importation of parasites into the country. Field workers based at the 13 regional health offices operated the screening program and were responsible for contacting passengers arriving from malaria endemic areas within 48 hours after arrival to collect a blood smear, household information, and recent travel history. The field workers then followed up with recurrent visits every 14 days for six weeks, each time taking a blood smear for diagnosis to the malaria-dedicated laboratory [8]. In 1969, 20,411 blood slides were taken from incoming passengers that arrived directly from malaria-endemic countries or those who had been in a malaria-endemic country within the previous six months [41]. Of these passengers, 92.6% were visited once by a field worker, but only 26.8% were monitored for the full 42-day surveillance period. On average, passenger screening alone – excluding other active case detection activities (i.e. fever surveys) and passive case detection – detected 43% of all positive malaria cases during POR [40]–[42].

### Reintroduction and elimination, 1975–1997

Local *P. vivax* transmission was reestablished in 1975 with an outbreak that began in a village just outside the capital, Port Louis, with 41 cases identified in a community of migrant workers [45]. The majority of these positive cases were found among workers from malaria-endemic India who came to help rebuild the island after considerable damage from a cyclone (Figure 1) [46]. From these initial cases, the outbreak increased to 668 cases and resulted in endemic transmission that continued for 23 years [28], [47]–[49]. The fact that the importation of parasites by migrant workers led to such resurgence is often attributed to new and ubiquitous breeding sites created by the results of the cyclone [38], [45]. In addition, however, it is important to consider two factors that may have contributed to resurgence: 1) lax interventions, including surveillance and vector control following the first elimination campaign, and 2) increased importation risk beyond that posed by migrant workers.

A WHO report noted that malaria-free certification in 1973 “was certainly responsible for a relaxation in case detection activities… And the integration of the malaria services into the preventive health services further contributed to the weakening of the surveillance mechanism,” [50] and the head of the Malaria Control Unit at the time agreed with these sentiments [38]. Surveillance and laboratory staff was deemed to be half of what the program required [50]. Cooperation with health workers was lacking completely—passive surveillance was virtually absent in 1975 with health workers unwilling to routinely test for malaria at health facilities [13]. Until a programmatic shift in 1982, it took nine days for blood smears taken in the field to be delivered to the malaria laboratory (while newspapers published in Port Louis reached the entire island in three hours) [51].

At the same time, the island's *An. arabiensis* density increased substantially as the presence of breeding sites multiplied, with the prevalence of anophelines indoors climbing from 0.4% in 1967 to 28% in 1972 [52]. These increases were likely due in part to cyclonic disruption, but may have also been the product of reduced vector control measures after malaria-free certification, especially IRS that was limited to only hundreds of households and ports of entry in the early 1970s [13]. In addition, it is possible that development contributed to this heightened receptivity: new housing structures with concrete flat rooftops—a reflection of major economic development at the time—were built throughout the island and contributed to increased vector density as pools of water conducive to anopheline breeding would collect and stagnate on these rooftops [53].

An increase in the rate of malaria importation may also have contributed to reemergence [13]. The number of arrivals steadily increased from 1933 but jumped substantially in the early 1970s with an almost fourfold increase from 1968 to 1975 [54], possibly due to increased demand for labor for a major economic development plan initiated in 1970 [55]. The majority of these visitors were from malaria-endemic areas, predominately mainland sub-Saharan Africa and India [56].

After the epidemic peaked in 1982 with 623 indigenous cases [57], local transmission was reduced to zero by 1990 with a combination of focal IRS, widespread larviciding, passenger screening, and an extensive case response system with every case parasitologically diagnosed by the reestablished malaria-dedicated laboratory [51]. Two small outbreaks in 1992 and 1996 followed, with the last indigenous case recorded in 1997 [12] as shown in Figure 1. Since 1998, Mauritius has maintained the absence of local transmission despite the continued presence of *An. arabiensis* and ongoing importation of malaria parasites [28].

### Prevention of reintroduction, 1998-present

The current POR program consists of three principal components that were in place in past programs but are arguably more robust today: first, the continuation of the passenger screening program; second, an Integrated Vector Management (IVM) strategy; and third, a strong health system that passively detects and responds to missed imported or introduced malaria cases.

The proactive passenger screening program traces an average of 175,000 incoming passengers arriving each year [28] who meet at least one of the inclusion criteria: traveling from malaria-endemic countries, report having been in a malaria-endemic country in the last six months, and/or those who report being febrile upon arrival [31] - the same criteria used since 1960. On average, 79% of passengers are contacted by visit or phone at least once by health surveillance officers (21% leave the country prior to contact or are untraceable) and 38% of passengers are under surveillance for the full 42-day surveillance period [28]. Longer surveillance is conducted for migrant workers since many originate from endemic areas, with health surveillance officers visiting monthly after the fourth formal follow-up visit for a total of three months [personal communication - Rawoteea]. Blood slides collected from these passengers are sent to the public malaria-dedicated laboratory, staffed by nine trained malaria microscopists, for diagnosis. Average annual blood examination rate (ABER) between 2005 and 2008 was 3.4% [28] (Figure 1). While there are more surveillance officers per 100,000 population compared to the first POR program, the number of officers per incoming passengers from malaria endemic regions has declined over time, as has the fraction of all cases detected through passenger screening—the first likely due to an increased number of arrivals each year and the latter potentially due to a strengthened passive surveillance system (Table 2).

**Table 2:** Surveillance indicators for active case detection.

	Elimination II^2^	POR I	POR II	
Indicator	*average 1982–88*	*1960–61*	*1990–91*	*2008*
# surveillance officer/incoming passengers from malaria endemic regions/1,000 population^1^	5.7	2.4	2.1	0.5
# surveillance officer/district	19.4	5.6	15	11.1
# surveillance officer/100,000 population	17.9	6.1	13.0	8.1
% positives detected by passenger screening	47.7	58.0	–	25.9

1 Extrapolated the number of passengers from endemic regions from 2005–2008 data.

2 The passenger screening program began after Elimination I during POR (Prevention of Reintroduction).

The current IVM strategy focuses on routine island-wide larviciding with temephos conducted in two-week cycles. IVM targets former malaria foci, breeding sites with high density of anopheles larvae identified during entomological surveillance, and high-risk areas around migrant workers' residences. New breeding sites are continuously identified by entomological surveillance and health inspections and are regularly added to the list for targeted larviciding. Individuals and villages are charged with the responsibility of looking after their environment and are managed by health inspectors who have Power of Entry granted by the country's Public Health Act of 1925 that permits them to legally require residents to remove breeding places in or around their residences [58].

Another integral component of Mauritius's IVM strategy is indoor and outdoor residual spraying at and around the port and airport with DDT or lambda cyhalothrin, a synthetic pyrethroid, every six months. Overall, national entomological surveillance data from 2004 to 2007 indicates zero presence of anophelines indoors and very low densities outdoors [59].

Passive case detection has also strengthened substantially compared to the first POR period as health workers have become increasingly participatory in screening patients and promptly sending blood smears to the malaria laboratory for diagnosis [60]. Passive case detection identifies an average of 47% of cases each year [28].

All slides are read by the malaria laboratory within 24 hours[personal communication – Lam], and identification of a malaria case leads to immediate treatment. Reactive case detection is triggered as part of an extensive case response system for all positive diagnoses and includes case investigation, contact tracing, and fever surveys. Larviciding and IRS, inspections of potential breeding sites, and health education of households on malaria risks to increase awareness and prevent further transmission are also conducted within a 500 meter radius of a case's residence and any other residence where the case stayed 18–24 days prior to diagnosis [61].

### Costs and capacity

**Figure 2:**
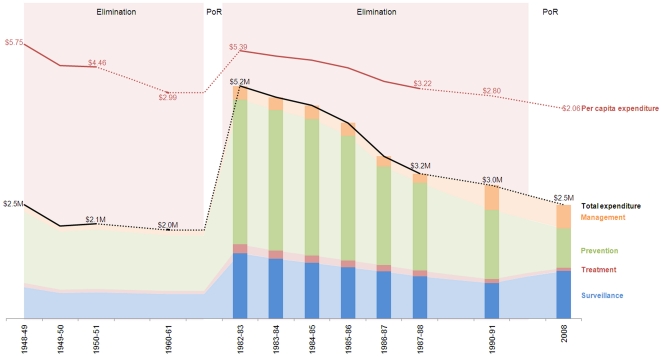
Total and per capita program costs, 1948–2008. *The bars reflect real data on expenditure per intervention while the lighter shading is extrapolated based on averages from 1982–1988. Literature indicates a similar allocation of funds, although surveillance-attributed expenditure was probably proportionally higher around 1960 due to a change of strategy with a new focus on surveillance. This figure indicates that the cost of malaria control dropped steadily since 1982, with per capita costs dropping faster than total costs due to growing population size (NB different vertical scales).

As described in Figure 2, annual per capita cost of the current POR program is $2.06 (in 2008 dollars) or 0.83% of public health expenditure, a significant reduction from costs during elimination and also lower than the $2.99 per capita spent during the first POR period (9% of public health expenditure). As described in the methods, because phases were defined by strategy and combination of interventions, 1960 costs are categorized as POR. Figure 2 also illustrates the strategic shift from prevention activities to surveillance that is represented by expenditure proportional to each intervention during the recent elimination and current POR periods. Surveillance comprised an average 28% of annual expenditure during elimination but now amounts to 42% of total annual costs while prevention-related costs (i.e. vector control, environmental management, IEC, and prophylaxis) declined from 63% of total expenditure during elimination to 34% during POR today. Per capita costs for passenger screening and vector control—the two primary interventions over time—during elimination were $1.19 and $1.57 and during POR are $0.70 and $0.62, respectively. The Mauritius government was and continues to be the primary funder, although the World Health Organization has been contributing financial and other resources since the 1960s.

Table 3 demonstrates that the current POR program spends proportionally more on personnel (90% of total expenditure) compared to earlier periods, which had nearly equivalent spending for consumables and personnel. Despite integration, this POR effort remains personnel intensive with close to 400 people spending a proportion of their time on malaria-related activities (or 274 FTEs) although this is less than was required for either elimination campaign. Skilled labor constituted an average 22% of the workforce during elimination compared to 61% during POR. Costs per FTE are highest during the current POR at approximately $9,000, which currently includes an approximately 100-person surveillance staff and 100-person vector control staff, all spending close to 100% of their time on malaria-related activities.

**Table 3:** Costs and capacity of workforce.

	Elimination I^1^	Elimination II	POR I^2^	POR II	
Expenditure category	*average 1948–51*	*average 1982–88*	*1960–61*	*1990–91*	*2008*
Personnel	46%	83%	51%	93%	90%
Consumables and equipment	54%	16%	49%	6%	10%
Total workforce(% skilled vs. % unskilled)	614 *(21% vs. 79%)*	1,338 *(23% vs. 77%)*	–	534 *(27% vs. 73%)*	384 *(61% vs. 39%)*
Number of FTEs^2^	614	684	–	465	274
FTE/100,000 population	132	69	–	45	24
Average annual expenditure per FTE	$1,673	$6,748	–	$6,403	$9,161

1 It was not possible to calculate full time equivalents (FTEs) for the first elimination period so the full staff was used. However, planning documents from the campaign indicate that most staff were engaged directly in the three year campaign.

2 While total expenditure for personnel was available for 1960–1961 in technical reports on the elimination program, exact figures for total workforce and FTEs were not available.

### Evaluating the impact of POR surveillance measures

Data on the number of malaria cases identified each year were obtained since 1948, but detailed records of the means of detection were only available for 2005–2008. Of the 36 cases identified on average annually over this 4-year period, an average of 9 cases per year (26%) were detected each year through examination of blood taken from incoming passengers, while an average of 17 cases per year (47%) were identified passively in public and private clinics, and an average of 10 cases per year (27%) were detected through reactive case detection. These 27 cases identified by passive or active case detection after they had arrived in the country thus indicate than an approximate minimum of 27/36 cases (74%) were missed by the passenger screening program.

Although each of the infections identified by screening will lead to some risk of onwards transmission, this risk is minimized assuming that cases were promptly identified and treated in line with established protocols. Beyond the 74% of cases missed by passenger screening but identified by other case detection means, however, it is probable that additional cases escaped detection by all of these procedures due to imperfect sensitivity of screening or testing algorithms and microscopy, and thus had greater opportunity to lead to local transmission. Given assumptions about the sensitivity of tests and the completeness of qualifying criteria, it was estimated that an average of 86% of infections were missed by passenger screening, with a 95% CI of 80–89% (more than 90% of this variance was related to the assumed sensitivity of microscopy). This high fraction of imported infections missed by passenger screening is largely attributable to the low testing rate: although 148,642 incoming passengers were visited by health surveillance officers in 2008, only 36,538 slides were taken (24.6%) as surveillance officers only test passengers who are symptomatic within the 42-day surveillance period.

Records of malaria cases identified annually since the second POR program began in 1998 indicate an average of 48 cases identified by all means of detection each year. Assuming the same case detection fractions as observed from 2005–2008, it may be estimated that an annual average of 12 of these cases were identified by passenger screening, 23 by passive case detection, and 13 by reactive case detection. If 86% of cases were missed by passenger screening, however, as calculated above, an estimated 74 infections escaped detection by passenger screening, of which only 36 were identified annually by passive and reactive case detection. It is thus estimated that about 38 cases (95% CI of 12–61 cases) are unidentified, or missed by all case detection activities. Dividing this figure by Mauritius's average population from 1998–2008 of 1.2 M produces an estimated importation of 0.03/1000/year (0.01–0.05), assuming that the 48 cases identified by surveillance activities contributed little additional risk or secondary transmission beyond these cases that were not identified. If passenger screening were halted, the 12 cases it currently identifies annually on average would additionally enter the island; with an estimated 49% subsequently identified by passive and reactive case detection, a modest increase in effective importation of approximately 6 infections per year could be expected.

**Figure 3:**
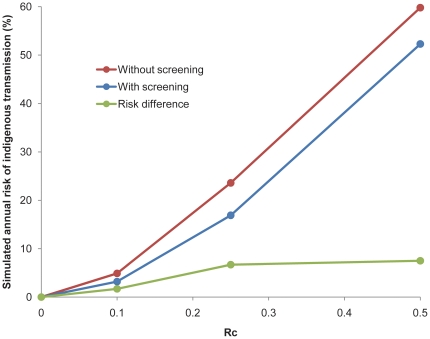
Simulated annual risk of indigenous transmission in Mauritius with and without passenger screening at a range of potential R_C_ values.

Simulations of potential malaria transmission using these importation rates and a range of R_C_ values indicate a current annual risk of secondary transmission of about 3% if R_C_ = 0.1, rising to 17% if R_C_ = 0.25 and 52% if R_C_ = 0.5 (Figure 3). Given the absence of local transmission in Mauritius over the past decade, the true value of R_C_ is thus most likely at the low end of this range. The increase in effective importation estimated to result from the cessation of the passenger screening would cause an accompanying increase in the annual risk of secondary transmission of 1.7% to 7.5%, depending upon the value assumed for R_C_.

## Discussion

Mauritius's initial failure and current success in preventing the reemergence of malaria provides a number of important lessons for the effective maintenance of elimination. Unlike in the first POR program, current efforts have succeeded in maintaining elimination despite large cyclones in 1994 and 2002 that caused costly damage ($81 million [62] and $50 million [63], respectively) and an increase in the number of travelers arriving from endemic countries [64]. Achieving this level of success has required substantial operational effort, including a large number of FTEs and a high level of sustained political and financial commitment. Part of this effort includes an extensive case response system to prevent introduced malaria cases following imported case detection, requiring rapid mobilization of resources and personnel at both district and national levels.

In part, the failure of the first POR program may be attributed to weakening of the surveillance system. The passenger screening system during the first POR program visited a higher percent of incoming passengers than today, but historical anecdotes indicate an underperforming program. The current program succeeds in contacting a higher fraction of travelers from malaria endemic countries repeated times, has improved operational capacity in districts, and more surveillance officers per 100,000 population (Table 1). The importation risk of 0.03 per 1,000 population per year estimated here is significantly lower than that estimated for the islands of Zanzibar – the only other known quantification of malaria importation [65]. Despite this relatively low risk, maintenance of the passenger screening program is most likely resulting in a reduction in risk of indigenous transmission of approximately 1.7%–7.5% each year; since R_C_ is assumed to be low, the lower end of this range is more likely. Although modest, the impact is significant once compounded over a decade (roughly 18% risk reduction) or longer, at a cost of $.70 per capita per year.

Until global eradication is achieved, most countries will always face some risk of malaria resurgence. The objective of a POR program in these countries is therefore not an absolute absence of malaria transmission, but rather an acceptable level of resurgence risk. To date, countries have set this objective implicitly, implementing interventions without a clear discussion of the baseline or targeted risk levels such as the passenger screening program in Mauritius. As more – and poorer – countries establish POR programs, this process should be formalized to ensure the most efficient interventions are pursued. Techniques to do so are commonly used in other fields and could be easily extended to malaria. For example, policies and interventions to prevent the introduction of zoonotic diseases and plant pests are designed based on a process known as import risk analysis, which centers on the country determining its baseline risk and acceptable risk target for each pathogen [66]. Although countries would ideally set risk targets through robust cost-benefit analysis, the complexity of this analysis may rely most on qualitative assessments of the implications of reintroduction of disease and the government's and public's attitude towards those potential outcomes [67].

Thus while Mauritius' passenger screening approach appears to be a major investment for relatively low impact at first glance, it may be acceptable spending if the country is risk averse. Mauritius has not formally set a risk target for its POR program; however, the government's actions since it achieved elimination the second time, including continued investment in the extensive surveillance program, indicates a low tolerance for risk. This suggests that the passenger screening program, with its moderate reduction in resurgence risk, is generally acceptable in the Mauritian context despite its relatively high costs and operational complexities. It is important, however, that other countries carefully assess this intervention in their own contexts before pursuing it as geographic realities (e.g., large land borders with malaria endemic areas) or higher risk tolerance (e.g., in poorer countries with many competing health priorities) would dramatically reduce its value. This assessment should ideally be part of a broader exercise to identify the most efficient set of interventions to achieve each country's risk target (e.g., using cost-minimization analysis) as other interventions such as improved passive surveillance may be more cost-effective.

Beyond surveillance, the Mauritius experience indicates that any expectation that spending can drop dramatically after elimination is achieved is erroneous and, in a highly vulnerable and receptive setting, could lead to rapid resurgence. Given that the isolated island of Mauritius spends substantially to maintain elimination, it is reasonable to expect that costs will be even higher in mainland countries with porous borders.

In a higher income country like Mauritius that spends US$247 per capita on health [68], $2 per capita to prevent reintroduction is viable. Mauritius's average annual per capita costs during elimination were nearly half that spent in Mayotte [69] and almost $4 less on POR than in Reunion Island [70]. Further comparisons of costs per phase are explained in more detail elsewhere [71]. In most sub-Saharan African countries however, where per capita expenditure is below US$100 and in some cases below US$20 [2], that investment seems more challenging to secure and maintain over a long period of time, especially once malaria is no longer perceived as a public health problem [72]. Mauritius has been financed almost entirely by domestic resources, with consistent funding ensure by strong political will; countries receiving substantial external funding may face greater challenges in securing the necessary sustained resources.

Despite these caveats, Mauritius demonstrates that it is possible to eliminate malaria and prevent its reintroduction with relatively high receptivity and vulnerability but that areas with high receptivity will likely need to maintain some form of control to reduce and sustain low vector density, as the country has exemplified by its ongoing larviciding and residual spraying activities. Evidence also suggests it may be necessary to maintain some malaria-specific capacity or a hybrid model of integration into the broader health system in order to sustain robust surveillance and vector interventions even though the WHO recommends full integration [73].

As proactive case detection is a major cost driver of a POR program and requires a large operational effort, there is a need for greater examination of the cost-effectiveness of this intervention. Although the current approach in Mauritius has successfully prevented reintroduction, there is a dearth of evidence on the design and cost of malaria surveillance to effectively prevent reemergence and a need to assess whether alternative strategies would be more cost-effective. This analysis on Mauritius's screening program is a first step, but a greater understanding of the most effective approaches to surveillance is needed as more countries design elimination and POR programs.

Notwithstanding these important lessons from Mauritius, the world knows disturbingly little about interventions to prevent the reemergence of malaria in resource-poor countries. With a growing volume of local and international resources allocated to achieving elimination, it is imperative that countries be in a position to sustain the benefits of that investment. To do so, they require a nuanced understanding of their risk of resurgence and the most cost-effective strategies to mitigate it. Additional robust research to develop this understanding should accordingly be a priority for institutions supporting elimination.
